# Adrenergic receptors on immune cells in cardiovascular disease: signaling plasticity, biased agonism, and therapeutic opportunities

**DOI:** 10.3389/fphys.2026.1792252

**Published:** 2026-05-14

**Authors:** Laurel A. Grisanti, Soraya Nekouian

**Affiliations:** 1 Department of Pathobiology and Integrative Biomedical Sciences, University of Missouri, Columbia, MO, United States; 2 Dalton Cardiovascular Research Center, University of Missouri, Columbia, MO, United States

**Keywords:** adrenergic receptors, cardiovascular disease, inflammation, neuro-immune, sympathetic nervous system

## Abstract

Sustained sympathetic nervous system activation is a hallmark of hypertension, atherosclerosis, ischemic injury, stroke, and heart failure. While adrenergic receptor (AR) biology is often discussed in cardiomyocytes, vascular smooth muscle, and endothelium, immune cells also express α_1_-, α_2_-, and β-ARs, enabling leukocytes to directly sense catecholamine tone and translate it into inflammatory or pro-resolving programs. In this review, we integrate subtype-specific signaling logic (α_1_AR-Gq/PLC/Ca2+; α_2_AR-Gi with reduced cAMP; βAR-Gs with cAMP-PKA/EPAC) with key regulatory features that determine outcomes *in vivo*, including receptor phosphorylation, trafficking and desensitization, compartmentalized signaling, and noncanonical pathways mediated by Gβγ and β-arrestins. We highlight how these mechanisms create context dependence across immune lineages and disease stages, shaping chemotaxis and adhesion, cytokine production, effector functions (for example ROS generation, antigen presentation, and cytotoxicity), and resolution programs such as IL-10-linked responses and efferocytosis. Across CVD contexts, immune cell AR signaling can amplify injury or constrain inflammation, thereby influencing endothelial dysfunction, vascular remodeling, fibrosis, microvascular impairment, and long-term organ function. Finally, we outline translational priorities, including improved mapping of AR expression and signaling states in human immune compartments, immune-phenotyped evaluation of AR-targeted therapies, and precision approaches using subtype-selective or biased ligands to modulate inflammation while preserving essential host defense and repair.

## 1 Introduction

### 1.1 Global burden of cardiovascular disease

Cardiovascular disease (CVD) remains the leading cause of morbidity and mortality worldwide, accounting for ~32% of global deaths and ~20 million deaths annually (Global Burden of Cardiovascular D. et al., 2025; Martin et al., 2025). In the United States, CVD caused 919, 032 deaths in 2023, with one person dying from CVD approximately every 34 seconds. Together, these data underscore the persistent global burden of CVD and the need to better define mechanisms that link neurohumoral stress, inflammation, and cardiovascular remodeling. Heart failure (HF), a complex syndrome encompassing both reduced ejection fraction (HFrEF) and preserved ejection fraction (HFpEF), remains associated with substantial morbidity and mortality. Hypertension, which affects nearly half of U.S. adults, is a major independent risk factor for HF and atherosclerotic vascular disease (Bozkurt et al., 2025; Martin et al., 2025).

### 1.2 Sympathetic nervous system activation and adrenergic signaling in cardiovascular disease

The sympathetic nervous system (SNS) mediates rapid adaptive responses to acute stress through catecholamine release, with norepinephrine (NE) released primarily from sympathetic nerve terminals and epinephrine (Epi) predominantly from the adrenal medulla (Godoy et al., 2018). In healthy physiology, acute SNS activation transiently increases vascular tone and peripheral resistance to maintain blood pressure homeostasis, while increasing heart rate and myocardial contractility to match metabolic demand (Hein and Kobilka, 1997; Lymperopoulos et al., 2013). These effects are mediated by adrenergic receptors (ARs), which are grouped into three receptor families—α1, α2, and β—each with three recognized subtypes (Hein and Kobilka, 1997). The roles of adrenergic signaling in vascular smooth muscle cells (VSMCs), endothelial cells (ECs), and cardiomyocytes are well established and account for many of the canonical cardiovascular effects of SNS activation in both physiological and disease settings (Hein and Kobilka, 1997; Wu et al., 2021). By contrast, adrenergic signaling in immune cells is less well defined than its canonical actions in vascular and cardiac cells (Sharma and Farrar, 2020; Chhatar and Lal, 2021). ARs expression has been reported across multiple immune cell populations, and catecholamines can modulate immune cell trafficking, activation, cytokine production, and remodeling-related responses (Grisanti et al., 2016a; Peklo et al., 2025). However, the magnitude, direction, and disease relevance of these effects are often context dependent and remain incompletely defined, varying by receptor subtype, cell state, tissue niche, and source of catecholamine exposure (Sharma and Farrar, 2020; Chhatar and Lal, 2021; Freire et al., 2022). Accordingly, in this review we distinguish established cardiovascular actions of adrenergic signaling from emerging immune-cell–centered mechanisms that are supported by experimental evidence but are not yet uniformly resolved across disease settings.

**Figure 1:**
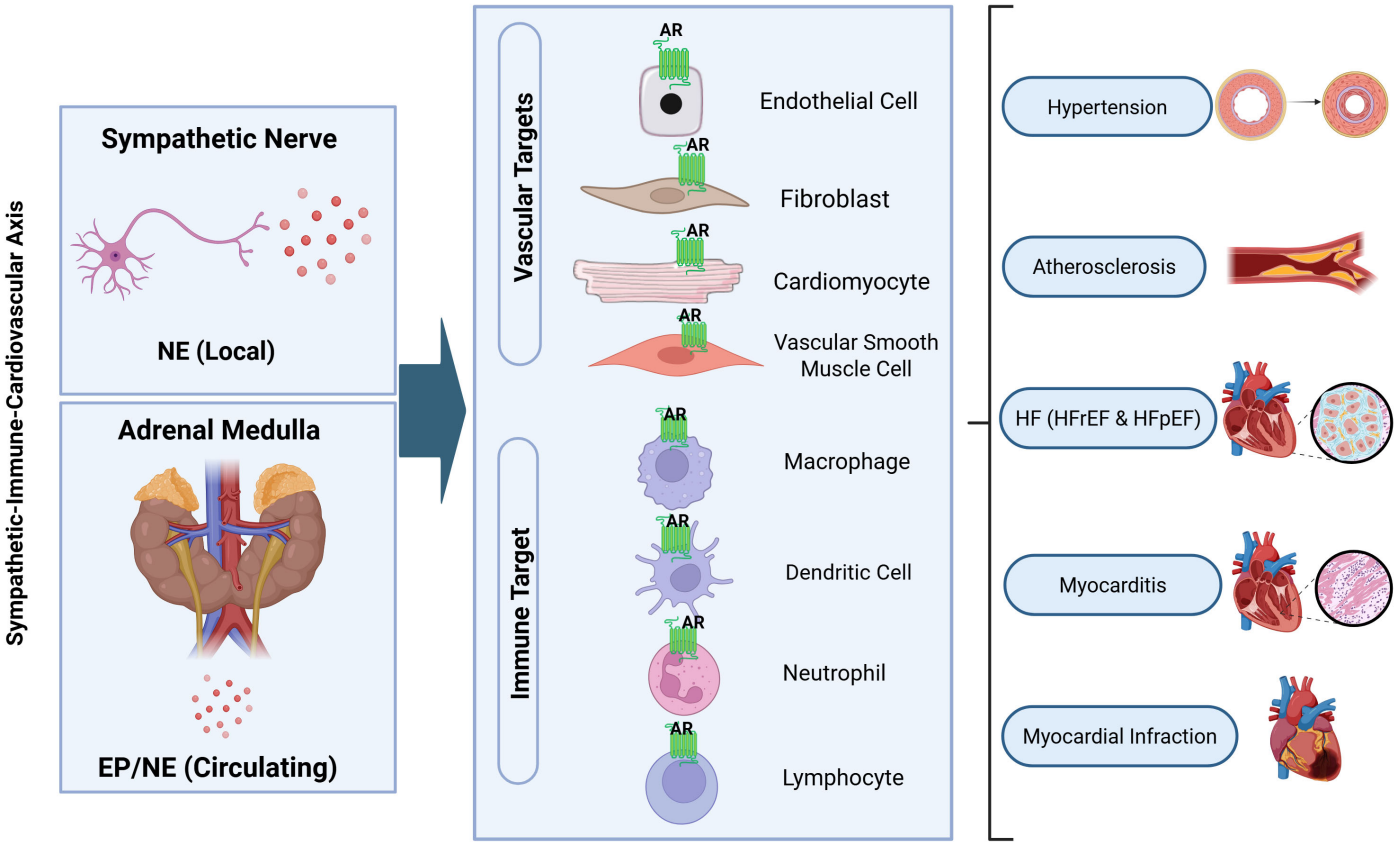
Overview of the sympathetic–immune–cardiovascular axis in cardiovascular disease. Sympathetic nerve terminals release norepinephrine (NE) locally within cardiovascular tissues, whereas the adrenal medulla releases epinephrine (Epi) and NE into the circulation, generating endocrine catecholamine signals. These catecholamines engage adrenergic receptors (ARs) expressed on major vascular targets—endothelial cells, fibroblasts, cardiomyocytes, and vascular smooth muscle cells—as well as on immune cell targets including macrophages, dendritic cells, neutrophils, and lymphocytes. By acting on both vascular and immune compartments, adrenergic signaling integrates neurohumoral stress with inflammation and remodeling across the disease contexts reviewed here, including systemic hypertension (HTN), pulmonary HTN, atherosclerosis, heart failure with reduced ejection fraction (HFrEF) and heart failure with preserved ejection fraction (HFpEF), myocarditis, and myocardial infarction.

An overview of the sympathetic–immune–cardiovascular axis reviewed here is shown in **Figure 1**, which provides a conceptual framework highlighting local norepinephrine release from sympathetic nerve terminals versus circulating adrenal catecholamines, the major vascular and immune cellular targets of adrenergic receptors, and the spectrum of CVD contexts discussed in this review.

## 2 Adrenergic receptor biology: subtypes and signaling logic

**Figure 2:**
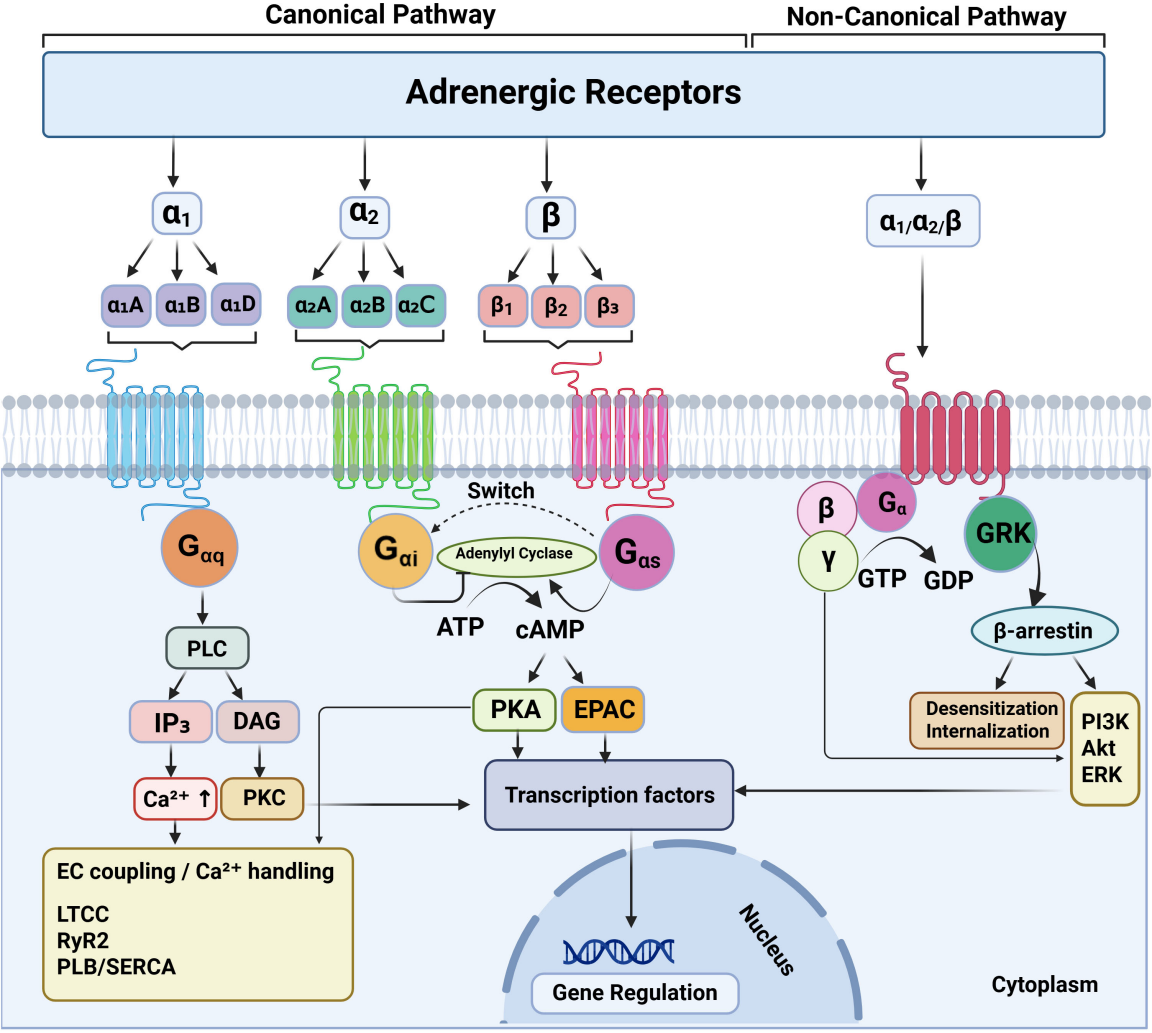
Adrenergic receptor subtypes and core signaling/regulatory logic relevant to cardiovascular and immune responses. Adrenergic receptors (ARs) are class A G protein–coupled receptors comprising three families—α_1_ (α_1_A/α_1_B/α_1_D), α_2_ (α_2_A/α_2_B/α_2_C), and β (β_1_/β_2_/β_3_)—that translate catecholamine cues into subtype-biased intracellular programs. α_1_ARs couple predominantly to Gαq/11 to activate phospholipase C (PLC), generating IP_3_ and DAG, increasing intracellular Ca^2+^, and activating protein kinase C (PKC). α_2_ARs preferentially couple to Gαi/o to inhibit adenylyl cyclase and reduce cAMP-dependent signaling, while released Gβγ subunits can engage additional downstream effectors. βARs classically couple to Gαs to stimulate adenylyl cyclase, increase cAMP, and activate protein kinase A (PKA) and exchange protein directly activated by cAMP (EPAC); depending on cellular context and receptor phosphorylation state, βARs—particularly β_2_AR—can also exhibit coupling plasticity, including switching from Gs to Gi and redirecting signaling toward alternative kinase pathways. Across AR families, receptor activation promotes G protein–coupled receptor kinase (GRK)-mediated phosphorylation, β-arrestin recruitment, and receptor desensitization/internalization. In addition to their role in desensitization, β-arrestins can scaffold signaling complexes that mediate G protein–independent outputs.

Adrenergic receptors (ARs) are class A G protein–coupled receptors (GPCRs) that translate catecholamine cues—NE and Epi, delivered in tonic or phasic patterns—into intracellular programs through subtype-selective coupling, regulatory phosphorylation, and trafficking. The three major receptor families—α_1_, α_2_, and β—share a canonical seven-transmembrane architecture but differ in preferred G protein-coupling, downstream effector engagement, and regulatory properties (Hein and Kobilka, 1997; Wu et al., 2021). In cardiovascular tissues, many of these signaling principles are well established, whereas in immune cells their functional consequences are supported by accumulating evidence but remain more context dependent (Sharma and Farrar, 2020; Chhatar and Lal, 2021). Canonical signaling logic for α_1_AR, α_2_AR, and βAR subfamilies, including subtype-selective G-protein coupling and regulation by GRK phosphorylation, β-arrestin recruitment, and desensitization/trafficking, are summarized in **Figure 2**.

### 2.1 Overview of adrenergic receptor subtypes

In immune cells, available evidence indicates that α1AR signaling can enhance inflammatory cytokine production and may be regulated by local inflammatory cues, but these effects are less well defined than in vascular tissues and appear to vary by immune cell type and activation state.

α_1_-adrenergic receptors (α_1_ARs) comprise three subtypes, α_1_A, α_1_B, α_1_D, and are classically coupled to Gαq/11, leading to activation of phospholipase Cβ (PLCβ) (Cotecchia, 2010; Perez, 2021). This process has been extensively characterized in vascular smooth muscle cells where it has been shown that PLCβ hydrolyzes phosphatidylinositol 4, 5-bisphosphate (PIP_2_) into inositol 1, 4, 5-trisphosphate (IP_3_) and diacylglycerol (DAG), leading to IP_3_-mediated Ca^2+^ release from intracellular stores and DAG-dependent activation of protein kinase C (PKC), which promotes vasoconstriction, medial hypertrophy, and increased stiffness (Chen and Minneman, 2005; Cotecchia, 2010). In immune cells, available evidence suggests that α_1_AR signaling in monocytes/macrophages can enhance inflammatory cytokine production and is shaped by local inflammatory cues, although these effects remain less well defined than in vascular tissues and depend on activation state (Heijnen et al., 2002; Grisanti et al., 2011b).

α_2_-adrenergic receptors (α_2_ARs) are further categorized into α_2_A, α_2_B, α_2_C subtypes and have been classically described as preferentially coupling to Gαi/o proteins. This signaling paradigm has been extensively described in the nervous system where Gαi signaling inhibits adenylyl cyclase, lowers intracellular cAMP, and reduces PKA activity, while Gβγ subunits modulate ion channels, vesicle fusion, and certain kinases (Wang and Limbird, 2007; Xu et al., 2022). Presynaptically, α_2_ARs act as inhibitory autoreceptors that limit NE release from sympathetic terminals, providing a critical negative-feedback mechanism on sympathetic tone (Miyamoto et al., 2008; Zhang et al., 2009). Postsynaptically, including on immune cells, α_2_ARs can influence chemotaxis, survival, cytokine and chemokine secretion, and integrin activation in a cell type– and context-dependent manner, often opposing or fine-tuning βAR-mediated cAMP signals (Scanzano and Cosentino, 2015; Chhatar and Lal, 2021). However, the extent to which these postsynaptic effects reflect direct immune cell–intrinsic signaling versus broader neural or tissue-level regulation is not always resolved (Chhatar and Lal, 2021).

β-adrenergic receptors (βARs) comprise three subtypes (β_1_, β_2_, β_3_) and are classically described as Gαs-coupled GPCRs that stimulate adenylyl cyclase, increase cAMP, and activate protein kinase A (PKA) and exchange proteins directly activated by cAMP (EPACs) (Lohse et al., 2003; Pandey et al., 2020). In the heart, β_1_AR is the dominant mediator of positive inotropy and chronotropy, whereas β_2_AR and β_3_AR contribute to both contractile regulation and cardioprotective or anti-remodeling programs in a context-dependent manner (Lohse et al., 2003; Lin et al., 2024). In the vasculature, βARs generally promote vasodilation in select beds and modulate endothelial function (Schindler et al., 2004; Greaney et al., 2022). In immune cells, β_2_AR is typically the most abundant subtype, with β_1_AR and β_3_AR expressed at lower levels or in subset-restricted patterns although the functional significance of lower-abundance receptor subtypes remains less consistently established across studies (Sanders, 2012; Chhatar and Lal, 2021). Because β_2_AR is the most consistently implicated subtype in immune-cell regulation, it is emphasized in the disease sections that follow, whereas α_1_AR and α_2_AR are discussed where evidence directly supports roles in vascular inflammation, leukocyte trafficking, or immune activation.

Beyond canonical Gαs–cAMP signaling, βAR activation engages parallel Gβγ- and β-arrestin–dependent pathways that can alter cell type–specific responses (Belmonte and Blaxall, 2011; Shenoy and Lefkowitz, 2011). Upon agonist binding and G-protein activation, liberated Gβγ dimers can recruit GRK2 and related kinases to the plasma membrane, promoting receptor phosphorylation, desensitization, and internalization, while also directly engaging downstream effectors (e.g., PI3K and select PLC/ion channel modules) that influence hypertrophic, survival, and metabolic signaling in cardiomyocytes and vascular cells (Casey et al., 2010; Kamal et al., 2014). Following GRK-mediated phosphorylation, β-arrestins not only sterically terminate further G-protein activation but also function as scaffolds for signalosomes (e.g., Src-, ERK/mitogen-activated protein kinase (MAPK)-, and transactivation modules), enabling G protein–independent signaling with distinct effects on contractility, gene expression, and remodeling in the heart and other adrenergic-responsive tissues (Lefkowitz and Shenoy, 2005; Shenoy and Lefkowitz, 2011). Together, these mechanisms provide a conceptual basis for how identical catecholamine cues can produce divergent outcomes depending on receptor subtype, cellular context, and signaling compartmentalization.

Importantly, βAR signaling can also exhibit coupling plasticity. β_2_AR (and in some contexts β_3_AR) can undergo “Gαs/Gαi switching, ” in which receptor phosphorylation state and ligand–receptor interactions favor coupling to Gαi rather than Gαs, redirecting signaling toward alternative pathways such as PI3K/Akt or ERK and reshaping functional outputs in both cardiovascular and immune compartments (Daaka et al., 1997; Magocsi et al., 2007). Collectively, βAR signaling through Gα, Gβγ, and β-arrestin creates multiple axes along which ligands can exhibit biased signaling—a concept that underlies β-arrestin–biased agonists and Gβγ-targeted strategies discussed in later sections (Lefkowitz and Shenoy, 2005; Shenoy and Lefkowitz, 2011). Taken together, this complexity demonstrates that adrenergic signaling is not uniform, and receptor subtype, cell type, anatomical niche, and duration of catecholamine exposure all influence whether signaling favors inflammatory activation, resolution, or maladaptive remodeling.

### 2.2 Chronic SNS dysregulation in CVD: vascular and cardiac maladaptation

#### 2.2.1 Vascular maladaptation

In CVD, SNS activation shifts from a transient homeostatic response to a sustained maladaptive program with particularly important consequences for the vasculature. In hypertension and atherosclerotic vascular disease, heightened sympathetic tone promotes sustained vasoconstriction, impaired endothelium-dependent vasodilation, vascular remodeling, and renal sodium retention (Grassi et al., 1998; Al-Sharea et al., 2019). Together, these processes reinforce elevated vascular resistance and contribute to a pro-inflammatory vascular milieu (Guzik et al., 2007; Al-Sharea et al., 2019). Even in ischemic heart disease, myocardial infarction (MI), and chronic HF, persistent neurohumoral activation propagates systemic and regional vascular dysfunction, including increased arterial stiffness, endothelial dysfunction, and maladaptive remodeling, which worsen perfusion and accelerate end-organ injury (Ramsey et al., 1995; Landmesser et al., 2002).

Mechanistically, prolonged adrenergic signaling in the vessel wall drives several of these maladaptive responses. In resistance arteries and conduit vessels, sustained α_1_AR-mediated signaling in VSMCs promotes medial hypertrophy, increased stiffness, and impaired vasodilatory reserve, while endothelial dysfunction limits nitric oxide bioavailability and favors a pro-inflammatory, pro-thrombotic state (Nishio et al., 1998; Landmesser et al., 2002). These vascular effects are predominantly linked to direct adrenergic signaling within the target tissue, especially local norepinephrine release from sympathetic fibers and circulating catecholamine exposure at the vessel wall. Dysregulated adrenergic signaling in vascular cells has likewise been implicated in pathological remodeling and impaired vasodilator capacity, consistent with the concept that receptor systems supporting acute adaptation become maladaptive when chronically engaged (Davel et al., 2014; Valovic et al., 2023).

Despite the clear clinical benefit of βAR antagonists in major cardiovascular indications, β-blockers do not fully normalize vascular inflammation, endothelial dysfunction, or long-term structural remodeling (CIBIS-II Investigators and Committees, 1999; Su, 2015). This gap has prompted growing interest in additional pathways through which sustained SNS activation continues to influence cardiovascular structure and function, particularly through its actions on vascular cells and, as discussed in later sections, on immune cells (Dutta et al., 2012; Davel et al., 2014). By contrast, effects on immune-cell production, mobilization, or phenotype may also arise indirectly through remote neuroimmune regulation in the bone marrow and spleen, a distinction that becomes especially important in the disease-specific sections that follow (Dutta et al., 2012).

#### 2.2.2 Cardiac maladaptation

Chronic SNS activation also has major maladaptive consequences in the heart. In ischemic heart disease, MI, and HF, sustained catecholaminergic drive acts together with other neurohumoral systems, including the renin–angiotensin–aldosterone system, to promote myocardial stress, adverse remodeling, fibrosis, and progressive functional decline (Landmesser et al., 2002; Liao et al., 2022). Although many of the hemodynamic consequences of chronic SNS activation are mediated through the vasculature, direct adrenergic signaling within the myocardium is also a central driver of disease progression. Chronic β_1_AR overstimulation in cardiomyocytes contributes to maladaptive intracellular signaling, contractile dysfunction, hypertrophic growth, and remodeling programs that ultimately impair cardiac performance (Landmesser et al., 2002; Holwerda et al., 2019). These cardiac effects are distinct from, but closely intertwined with, vascular maladaptation: persistent increases in afterload, arterial stiffness, and endothelial dysfunction further amplify myocardial stress and accelerate HF progression. Thus, chronic catecholaminergic drive should be viewed not as an isolated myocardial signal, but as part of a broader cardiovascular program in which vascular and cardiac dysfunction reinforce one another.

At the same time, the contribution of chronic adrenergic signaling in nonmyocyte stromal and immune compartments of the heart remains less clearly resolved than its role in cardiomyocytes and vascular cells. This uncertainty is especially relevant because immune and stromal responses can influence fibrosis, repair, and long-term remodeling, yet the extent to which these effects reflect direct cardiac neuroimmune signaling versus indirect systemic regulation remains an active area of investigation (Dutta et al., 2012). Overall, the cardiac literature strongly supports maladaptive effects of chronic SNS activation on myocardial structure and function, but the vascular contribution remains better established as the primary system-level effector linking persistent sympathetic drive to long-term increases in afterload and impaired tissue perfusion.

### 2.3 Inflammation in CVD and immune cells as adrenergic targets

Inflammation is not merely a bystander in CVD; it is a mechanistic contributor to disease initiation, propagation, and clinical events across both vascular and cardiac syndromes (Tardif et al., 2019; Nidorf et al., 2020). In atherosclerosis, endothelial activation and leukocyte recruitment sustain plaque inflammation, promote necrotic core expansion and fibrous cap weakening, and increase susceptibility to plaque disruption and thrombosis (Cybulsky and Gimbrone, 1991; Dawson et al., 1999). In MI, the inflammatory response is required for debris clearance and scar formation, but excessive or prolonged innate activation can impair resolution, exacerbate adverse remodeling, and increase risk of HF progression (Dewald et al., 2005; Nahrendorf et al., 2007). In chronic HF, both innate and adaptive immune programs, including monocyte/macrophage activation, T-cell and B-cell responses, and cytokine/chemokine networks, contribute to fibrosis, cardiomyocyte dysfunction, and systemic inflammation that tracks disease severity and outcomes (Zouggari et al., 2013; Bansal et al., 2017). In hypertension and vascular aging, inflammatory signaling within the vessel wall promotes endothelial dysfunction, increased stiffness, and remodeling that reinforce elevated resistance and end-organ injury (Guzik et al., 2007; Madhur et al., 2010).

These inflammatory mechanisms operate through discrete cellular functions, including endothelial adhesion molecule expression and barrier changes, leukocyte trafficking and cytokine production, macrophage polarization and efferocytosis, antigen presentation and adaptive immune activation, and fibroblast/myofibroblast-driven extracellular matrix deposition, that collectively shape remodeling and long-term organ dysfunction (Sage et al., 2014; Khalil et al., 2017). Importantly, many of these steps are sensitive to neurohumoral cues. Sympathetic neurotransmission and AR signaling can modulate leukocyte mobilization, recruitment, activation state, and effector programs, creating a mechanistic rationale for examining adrenergic regulation within specific immune cell populations in CVD (Dutta et al., 2012; Agac et al., 2018).

Within this inflammatory framework, a key conceptual advance has been the recognition that immune cells are themselves direct targets of catecholamines. Over the past two decades, pharmacologic studies, genetic models, and transcriptional profiling have identified functional AR expression across multiple immune lineages, supporting the idea that sympathetic inputs can shape immune-cell trafficking, activation thresholds, cytokine programs, and survival (Madden et al., 1995; Bucsek et al., 2018). At the same time, the strength of evidence is not uniform across immune populations or experimental systems. Transcript detection does not necessarily predict surface protein abundance or functional coupling, GPCR antibody-based detection can be confounded by nonspecific reagents, and pharmacologic agonist or antagonist responses do not by themselves establish receptor subtype engagement, particularly when ligands have off-target activity (Hamdani and van der Velden, 2009; Schwanhausser et al., 2011). *In vivo* systemic manipulations add another layer of complexity because altered immune phenotypes may reflect indirect effects mediated through hemodynamics, neuronal circuits, endocrine responses, or stromal compartments rather than immune cell–intrinsic signaling (Katayama et al., 2006; Mendez-Ferrer et al., 2008).

Accordingly, the most persuasive conclusions are those supported by orthogonal approaches, including validated receptor detection, subtype-aware pharmacology with appropriate controls, and immune cell–specific genetic strategies, together with explicit attention to species and model limitations and to disease-stage context (Hamdani and van der Velden, 2009; Felce et al., 2020). Taken together, current evidence supports immune cells as adrenergic-responsive populations, but the degree of receptor expression, subtype dominance, and functional significance remains better established in some immune lineages than in others (Chhatar and Lal, 2021). This framework provides the basis for the immune cell–specific sections that follow.

## 3 Expression of adrenergic receptors on immune cell subsets

### 3.1 Myeloid cells (monocytes, macrophages, dendritic cells, neutrophils)

Macrophages are present in cardiovascular tissues at baseline and include resident populations that continuously survey the local environment and rapidly respond to injury cues (Epelman et al., 2014; de Couto, 2019). After acute vascular or myocardial damage, early macrophage- and stromal-derived mediators promote endothelial activation and establish chemokine gradients that drive recruitment of circulating leukocytes (Dewald et al., 2005; de Couto, 2019). Neutrophils are typically among the first recruited cells and can amplify inflammation through proteases, ROS, and NETs, while also shaping subsequent monocyte recruitment and macrophage differentiation through cytokines, DAMP release, and effects on endothelial activation (Horckmans et al., 2017; Ma, 2021). In many models, inflammatory monocytes (e.g., Ly6C^high/CCR2^+) accumulate next and differentiate into macrophages that initially support clearance/inflammatory programs and then transition toward reparative/resolving states (efferocytosis, IL-10/TGF-β, ECM organization) as the tissue progresses toward healing (Nahrendorf et al., 2007; Hilgendorf et al., 2014). Dendritic cells participate throughout but are especially important for antigen presentation and the later emergence of adaptive immune programs by shaping T-cell priming in draining lymphoid tissues (Choo et al., 2017; Forte et al., 2021). This sequence provides the main framework needed to interpret adrenergic regulation in myeloid cells across cardiovascular injury and remodeling states (Dutta et al., 2012).

Innate myeloid cells express a broad complement of ARs, with β_2_AR and α_2_AR particularly well documented on circulating monocytes and tissue macrophages in both mice and humans (Ignatowski et al., 2000; Peklo et al., 2025). Transcriptional profiling, radioligand binding, and functional assays have consistently shown that β_2_AR is the dominant adrenergic subtype under homeostatic conditions, while α_2_AR and, in some settings, α_1_AR are induced or upregulated in response to inflammatory cytokines, hypoxia, or microbial products (Heijnen et al., 2002; Peklo et al., 2025). In most settings, β_2_AR–Gαs–cAMP–PKA signaling in monocytes/macrophages suppresses production of pro-inflammatory cytokines such as TNF and IL-6, enhances rapid IL-10 secretion, and can reduce phagocytic “burst” and antigen-presenting capacity (Grailer et al., 2014; Ng and Toews, 2016). Functionally, these effects are generally consistent with limitation of excessive early inflammatory amplification and with support of the later shift toward reparative, pro-resolving macrophage states that support resolution and tissue repair (Grailer et al., 2014; Kourtzelis et al., 2019). Because macrophage cytokine output and efferocytosis efficiency strongly influence neutrophil persistence and the magnitude of continued monocyte recruitment, adrenergic tuning of macrophage programs may indirectly influence the duration and intensity of downstream waves (Kourtzelis et al., 2019; Tanner et al., 2021).

Two contextual variables are especially important. First, catecholamine source differs by anatomical niche. Myeloid cells may encounter direct local NE release within diseased tissues or perivascular compartments, circulating catecholamines in blood, or indirect neuroimmune regulation through bone marrow and splenic reservoirs before entering target tissues (Swirski et al., 2009; Dutta et al., 2012). Second, receptor responsiveness can change over time during chronic inflammation through desensitization, altered receptor balance, and shifting downstream pathway use (Ng and Toews, 2016; Freire et al., 2022). These factors likely explain much of the variability across studies and are more directly relevant to later disease sections than a broader catalog of downstream signaling branches. α_2_AR engagement, through Gαi-dependent pathways, decreases cAMP and modulates MAPK and calcium-sensitive signaling in ways that can influence chemotaxis, survival, and phagocytic activity in a context-dependent manner (Ignatowski et al., 2000; Miksa et al., 2009). As a result, the net catecholaminergic effect on monocytes and macrophages often reflects the balance of β_2_ versus α_2_ input, the prevailing cytokine milieu, and whether sympathetic activation is phasic or sustained (Wahle et al., 2005; Ng and Toews, 2016). In vascular disease settings, including atherosclerotic plaque inflammation and hypertensive vascular remodeling, myeloid-intrinsic adrenergic signaling has been implicated in regulation of trafficking and retention programs (including chemokine receptor axes such as CCR2 and CXCR4), thereby shaping the timing and magnitude of Ly6C^high monocyte recruitment to diseased vessels and their transition into inflammatory versus reparative macrophage states (Boring et al., 1998; Guzik et al., 2007). In the post-MI heart, where sympathetic drive and monocyte recruitment are both intense, immune cell-intrinsic adrenergic signaling has likewise been shown to regulate CCR2, CXCR4, and related trafficking receptors and to control the timing and magnitude of Ly6C^high monocyte influx and subsequent macrophage transitions (Grisanti et al., 2016a; Grisanti et al., 2016b). Related principles are likely relevant in pressure-overload HF as well but remain to be experimentally tested (Revelo et al., 2021; Tanner et al., 2021).

Neutrophils express functional adrenergic receptors, with β_2_AR representing the best-established subtype; evidence for α_1_AR involvement is more limited and appears to be context dependent (de Coupade et al., 2004; Scanzano and Cosentino, 2015). β_2_AR stimulation in neutrophils is commonly associated with reduced chemotaxis, ROS production, and NET formation, consistent with cAMP-linked inhibition of cytoskeletal remodeling and NADPH oxidase activity, and with reduced collateral tissue injury during acute inflammation (Silvestri et al., 1999; Marino et al., 2018). Because neutrophil-derived mediators influence endothelial activation and monocyte recruitment, adrenergic restraint of neutrophil activation early after injury could indirectly limit the size of the subsequent monocyte/macrophage wave (Ishihara et al., 2006; Wantha et al., 2013). This point is most relevant to acute injury settings such as MI and to vascular inflammatory states in which neutrophil-driven endothelial activation contributes to ongoing leukocyte recruitment. Because neutrophils are highly sensitive to rapid GPCR desensitization and receptor internalization, sustained catecholamine exposure can change responsiveness over time, contributing to variability across models that differ in disease stage and neurohumoral tone (Davies and Lefkowitz, 1981; Davies and Lefkowitz, 1983).

Dendritic cells (DCs) express β_2_AR and, to a lesser extent, αARs (Maestroni, 2000; Maestroni and Mazzola, 2003). In many systems, NE or β_2_-selective agonists shift DCs toward a more regulatory phenotype, reducing IL-12/IL-23, altering co-stimulatory molecule expression (CD80/CD86/CD40), and increasing IL-10 in a stimulus- and tissue-dependent manner (Maestroni and Mazzola, 2003; Nijhuis et al., 2014). These DC changes translate into altered T-cell priming that often reduces Th1/Th17 polarization and can enhance induction of Foxp3^+^ Tregs or IL-10^+^ regulatory programs, particularly in lymphoid tissues draining cardiovascular beds and in perivascular immune niches (Kim and Jones, 2010; Takenaka et al., 2016). Because DC programming sits at the innate to adaptive transition, adrenergic effects on DC cytokines and co-stimulation can have outsized downstream consequences for when and how strongly T-cell responses emerge (Maestroni and Mazzola, 2003; Takenaka et al., 2016).

Reported α and βAR expression in myeloid populations is frequently activation- and tissue-dependent, and conclusions based solely on transcript levels or non-validated antibodies should be interpreted cautiously (Herrera et al., 2013; Stoeckius et al., 2017). *In vivo* pharmacology additionally risks indirect effects through vascular or neurohumoral pathways, so the most persuasive evidence combines validated receptor detection with myeloid-intrinsic perturbation approaches and careful attention to disease stage and tissue niche (Katayama et al., 2006; Grisanti et al., 2016b).

### 3.2 Lymphocytes: T cells (CD4^+^, CD8^+^, Tregs)

T-cell involvement often becomes more apparent after the initial innate phase, consistent with the need for antigen capture/processing and priming (frequently by DCs and macrophages) before robust clonal expansion and tissue homing (Pasare and Medzhitov, 2004; Yan et al., 2013). Early innate cytokines and chemokines help set the trajectory by shaping antigen-presenting cell activation state and the balance of co-stimulatory versus regulatory signals, which in turn influences whether later T-cell accumulation amplifies injury/remodeling or supports resolution (Trevejo et al., 2001; Pasare and Medzhitov, 2004). Once present, T-cell cytokines can feed back onto vascular cells and myeloid populations, reinforcing endothelial activation and chemokine gradients (promoting continued recruitment) or restraining inflammation (limiting further influx and supporting repair), making timing and crosstalk central to interpreting adrenergic effects (Kassan et al., 2011; Wojkowska et al., 2017).

Among lymphocytes, T cells show prominent β_2_AR expression, with both CD4^+^ and CD8^+^ subsets typically expressing β_2_AR as the dominant AR subtype under many conditions (Williams et al., 1976; Anstead et al., 1998). β_2_AR density is dynamic and subset-associated, often low in naïve cells and modulated by activation and differentiation state, with differential maintenance across effector, memory, and tissue-resident compartments (Ramer-Quinn et al., 1997; Slota et al., 2015). In *in vitro* and ex vivo T-cell activation systems, including TCR-stimulation settings, β_2_AR engagement suppresses TCR-driven proliferation and effector cytokine production, with relatively strong inhibition of Th1-type (IFN-γ, TNF) and Th17-type (IL-17A/F) outputs and relative sparing or enhancement of Th2 and regulatory programs, in part through effects on transcriptional networks (T-bet, RORγt, GATA-3, Foxp3) and cytokine milieus (Ramer-Quinn et al., 1997; Liu et al., 2018).

Interpretation of T-cell adrenergic signaling is strengthened by explicitly considering stage and receptor plasticity. During priming and early activation, cAMP-linked signaling can dampen proximal activation outputs, whereas at later stages adrenergic cues can more strongly influence trafficking, tissue retention, and the balance between effector persistence and regulatory restraint (Vang et al., 2003; Nakai et al., 2014). Chronic catecholamine exposure, as occurs in sustained CVD neurohumoral activation, can remodel responsiveness through GRK/β-arrestin–dependent desensitization and trafficking, and potentially through coupling plasticity that shifts which downstream branches dominate over time (Ungerer et al., 1993). These stage-dependent effects likely matter most in hypertension, atherosclerosis, MI, and chronic HF, which differ substantially in duration and inflammatory architecture.

Because Th1/Th17 cells are implicated in vascular inflammation and remodeling in atherosclerosis, hypertension, and related syndromes, β_2_AR signaling places adrenergic tone in a position to influence whether CD4^+^ responses bias toward pathogenic versus resolving trajectories (Madhur et al., 2010; Smith et al., 2010; Chen et al., 2022). In cardiovascular models, immune-cell β_2_AR signaling has been linked to altered leukocyte recruitment and remodeling after MI and during chronic catecholamine stress, whereas studies in T cells outside CVD indicate that β_2_AR activation can suppress effector cytokine programs, including IFN-γ and, in some contexts, IL-17 (Sanders, 2012; Grisanti et al., 2016a; Grisanti et al., 2016b; Tanner et al., 2021). Beyond differentiation, β_2_AR activation can enhance CCR7- and CXCR4-mediated retention signaling and thereby reshape T-cell localization across lymphoid tissues and inflamed sites (Nakai et al., 2014). Where these effects arise from direct catecholamine exposure in target tissues versus indirect neuroimmune conditioning in lymphoid organs is not always resolved and should be distinguished in disease-specific sections whenever possible (Kohm and Sanders, 2001; Nakai et al., 2014; Thapa and Cao, 2023).

Regulatory T cells (Tregs) can become especially influential as inflammation persists, because their suppressive programs limit continued recruitment and help terminate cytokine-driven amplification loops. β_2_AR engagement has been linked to enhanced suppressive capacity, IL-10/TGF-β production, survival, and in some contexts tissue residency (Guereschi et al., 2013; Lu et al., 2023; Liu et al., 2025). Whether these effects remain sustained during chronic HF-like neurohumoral activation is less clear, because prolonged catecholamine exposure and receptor desensitization may attenuate these beneficial effects over time, which is relevant when comparing short-term stimulation experiments to chronic disease settings (Ungerer et al., 1993; Tanner et al., 2021).

Much of the foundational T cell–adrenergic literature predates modern definitions of helper and regulatory subsets, and because β_2_AR effects depend strongly on activation state and microenvironment, results from immortalized lines or highly artificial stimulation conditions may not fully recapitulate primary *in vivo* behavior (Williams et al., 1976; Bartelt et al., 2009). Lower-level expression of α_2_AR and β_1_/β_3_AR has been described in select T-cell contexts, and α_2_AR–Gαi signaling has been proposed to counterbalance β_2_AR–cAMP effects during chronic inflammation, but definitive *in vivo*, T cell–intrinsic evidence in CVD remains limited (Chhatar and Lal, 2021; Globig et al., 2023).

### 3.3 B cells

B-cell contributions often become more prominent as immune activation persists beyond the earliest innate phase, when antigen availability and T-cell help support germinal center activity, affinity maturation, and class switching. However, B cells can also shape earlier phases indirectly by modulating antigen presentation and cytokine environments that influence T-cell polarization and myeloid activation (Zouggari et al., 2013). Depending on subset composition, B-cell outputs can either reinforce chronic inflammatory recruitment loops (via pro-inflammatory cytokines and co-stimulation) or promote resolution (via IL-10–producing regulatory B cells), making them well positioned to influence whether inflammation transitions toward repair versus chronic remodeling (Barr et al., 2012; Jiao et al., 2021).

B lymphocytes express ARs, most prominently β_2_AR and, in several contexts, α_2_AR, allowing catecholamines to directly regulate humoral immunity (Pochet et al., 1979; Sanders, 2012). β_2_AR activation can modulate BCR signaling thresholds, antigen presentation, and co-stimulatory molecule expression (CD80/CD86/CD40), influencing germinal center dynamics including selection stringency, class-switch recombination, and differentiation toward plasma cells, memory B cells, or IL-10–producing regulatory B cells (Kohm et al., 2002; Podojil et al., 2004). In some models, β_2_AR signaling skews class-switching toward IgG1 and IgE, alters Tfh–B cell interactions, and favors emergence of Bregs, thereby shifting the balance between pathogenic antibody responses and regulatory humoral outputs (Kasprowicz et al., 2000; Honke et al., 2022).

For this review, the most relevant B-cell question is not broad humoral regulation in general, but whether adrenergic signaling shifts the balance between inflammatory, autoreactive, and regulatory B-cell programs in cardiovascular disease. As in other immune lineages, B-cell adrenergic effects are shaped by activation state and microenvironment, and likely by receptor regulatory dynamics during prolonged catecholamine exposure (Sanders, 2012; Honke et al., 2022). These effects may reflect direct catecholamine exposure in lymphoid or cardiovascular tissues, but they may also arise indirectly through neuroimmune control of splenic or marrow compartments before B cells enter diseased tissues. Because B-cell signaling depends on compartmentalized second messenger and MAPK microdomains, changes in receptor density, trafficking, and downstream pathway weighting could reprogram antigen presentation and cytokine output during chronic disease, although this remains incompletely tested *in vivo* in CVD models (Depoil et al., 2008; Barwick et al., 2016). Epigenetic regulation provides an additional potential layer of control, with AR expression and responsiveness potentially linked to differentiation state and tissue niche, but mechanistic evidence remains uneven across B cell subsets (Sanders, 2012; Barwick et al., 2016).

B-cell subsets differ markedly in antigen presentation capacity, cytokine competence, and regulatory potential, so bulk measurements can mask divergent subset-specific adrenergic effects (Attanavanich and Kearney, 2004; Yanaba et al., 2008). Where possible, conclusions are strongest when anchored to defined B-cell subsets and supported by validated receptor readouts and cell-intrinsic perturbation approaches. IL-10–producing B cells (“B10” cells) can be promoted by β_2_AR–cAMP signaling under certain stimuli, whereas α_2_AR engagement and/or chronic catecholaminergic environments have been associated with pro-inflammatory B-cell outputs (e.g., IL-6, GM-CSF) that can amplify myeloid activation and support Th17 programs (Li et al., 2015; Honke et al., 2022). This duality is conceptually important for CVD, where B cells contribute to remodeling and dysfunction through cytokines, antigen presentation, and antibodies (Adamo et al., 2020; Jiao et al., 2021).

In CVD, B cells are increasingly recognized as active modulators rather than passive antibody factories (Zouggari et al., 2013; Adamo et al., 2020). They contribute to experimental hypertension and vascular injury, influence post-MI remodeling and scar composition, and participate in HF and cardiomyopathies where autoantibodies against β_1_AR and other cardiac antigens are common and potentially pathogenic (Dimayuga et al., 2002; Jahns et al., 2004; Chan et al., 2015). Although direct *in vivo* evidence is still developing, sustained sympathetic activation and altered β_2_/α_2_AR signaling in B cells may influence germinal center selection and class switching, may affect survival of autoreactive clones, and may alter the balance between Bregs and inflammatory B-cell subsets in chronic pressure-overload and HF contexts (Kasprowicz et al., 2000; Honke et al., 2022).

### 3.4 Innate lymphoid cells and NK cells

NK cells and ILCs are adrenergic-responsive lymphoid populations, and available studies suggest that β_2_AR signaling can influence IFN-γ production, cytotoxicity, and type 2 cytokine programs (Moriyama et al., 2018; Wieduwild et al., 2020). However, compared with myeloid cells, T cells, and B cells, cardiovascular evidence in these populations remains more limited and less central to the main disease sections emphasized in this review. We therefore discuss NK cells and ILCs only briefly here. Existing work suggests that adrenergic regulation of these populations may be relevant to vascular inflammation, tissue repair, and stromal remodeling, particularly in perivascular niches, but mechanistic resolution in CVD remains incomplete (Yu et al., 2021; Chen et al., 2025).

## 4 Adrenergic regulation of immune responses across cardiovascular diseases

### 4.1 Hypertension and vascular remodeling

Hypertension is increasingly viewed as a chronic immune-driven vascular disease in which sympathetic activation intersects with leukocyte recruitment, endothelial dysfunction, and remodeling. In angiotensin (Ang) II- and deoxycorticosterone (DOCA)–salt–induced hypertension, macrophages and T cells accumulate in perivascular adipose tissue, the vascular wall, kidney, and heart, producing ROS and cytokines that drive vascular injury (Guzik et al., 2007; Marvar et al., 2010). SNS activation is strongly integrated into this process. Ang II stimulates both renin–angiotensin and sympathetic pathways, while catecholamines modulate immune-cell behavior through ARs (Marvar et al., 2010; Carnevale et al., 2016). In hypertension, adrenergic signals can arise from at least three sources: direct local NE release from sympathetic fibers innervating resistance arteries and perivascular adipose tissue; circulating catecholamines; and indirect neuroimmune regulation through hematopoietic reservoirs such as the bone marrow and spleen (Carnevale et al., 2016; Al-Sharea et al., 2019). Distinguishing among these routes is important because local vascular neuroimmune signaling should not automatically be interpreted as equivalent to systemic catecholamine exposure or remote leukocyte mobilization.

α_1_AR and β_2_AR on myeloid cells and T cells have been implicated in regulating vascular inflammation by controlling endothelial adhesion molecule (VCAM-1, ICAM-1) and chemokine (CCL2, CXCL9/10) expression, orchestrating perivascular accumulation of macrophages and effector/memory T cells (Grisanti et al., 2011b; de Juan et al., 2019). These immune cells produce IL-17, IFNγ, TNF, and ROS, which promote endothelial dysfunction, vascular stiffness, and media thickening (Guzik et al., 2007; Schuler et al., 2019). Sympathetic fibers innervating resistance arteries and perivascular fat therefore form with plausible local neuroimmune interfaces in which direct neural NE may amplify vascular inflammation (de Juan et al., 2019). Disrupting sympathetic input or AR signaling in these compartments can reduce vascular inflammation and blood pressure in some models, supporting the concept that immune-cell AR signaling can link hemodynamic stress and vascular remodeling (Carnevale et al., 2016; de Juan et al., 2019). By contrast, effects on leukocyte production and sustained recruitment may also be shaped indirectly through sympathetic regulation of marrow and splenic reservoirs (Carnevale et al., 2016). Beyond receptor-level changes, the anatomical sites of neuroimmune coupling (e.g., perivascular adipose tissue, kidney, and hematopoietic reservoirs) likely influence how sympathetic activation sustains leukocyte production and vascular infiltration over time (Carnevale et al., 2016; Xiao et al., 2020). Establishing whether and how immune-cell AR desensitization occurs in hypertension will require longitudinal, immune-cell–specific approaches paired with functional readouts (Mausbach et al., 2008).

**Figure 3:**
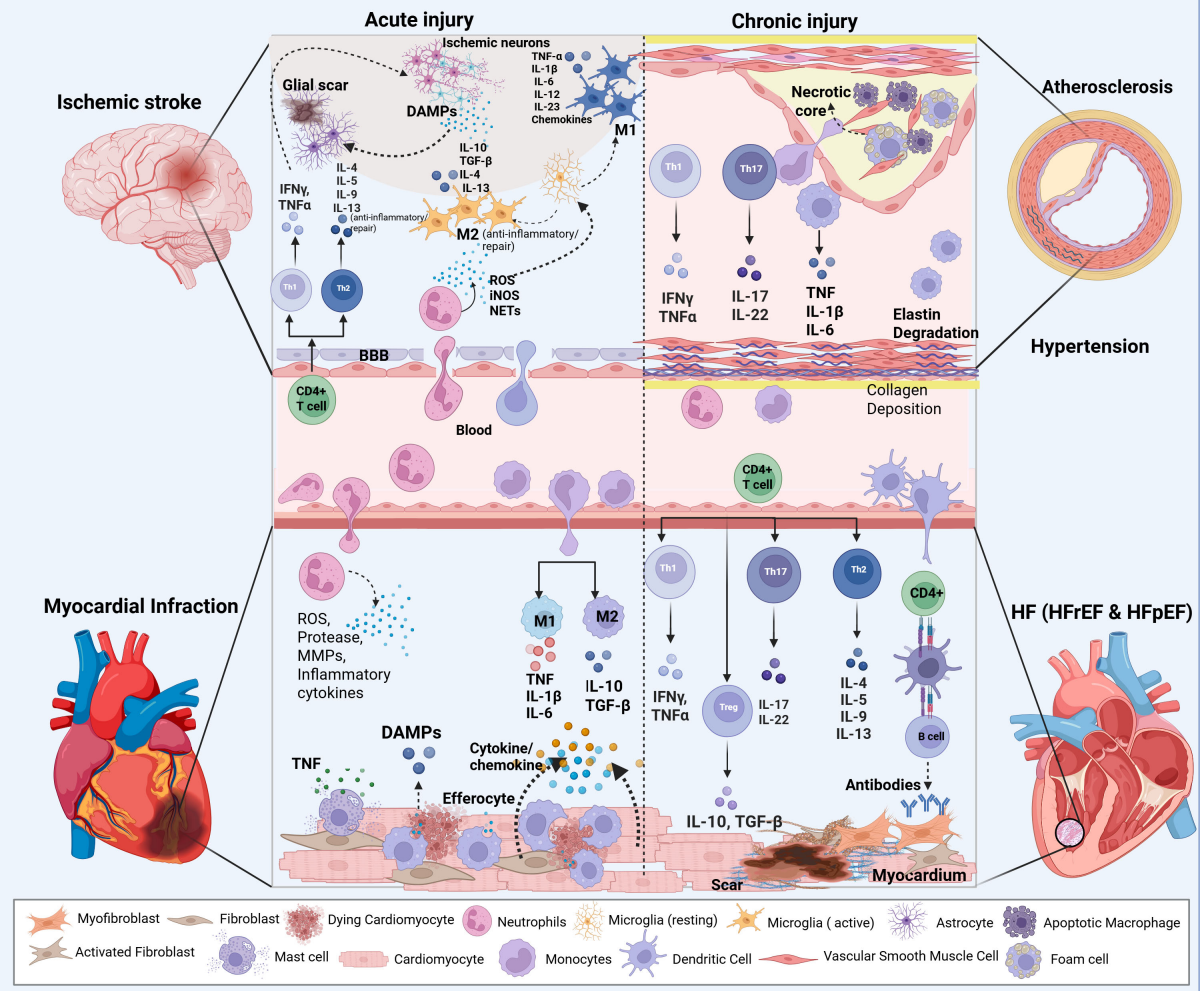
Shared immune effector pathways linking acute injury to chronic inflammatory remodeling across cardiovascular disease contexts. The schematic contrasts acute injury (left) and chronic injury (right) programs across the disease settings discussed in this review—myocardial infarction, atherosclerosis, hypertension, and heart failure with reduced ejection fraction (HFrEF) or preserved ejection fraction (HFpEF). In acute injury (myocardial infarction), sterile damage-associated molecular patterns (DAMPs) and early endothelial activation promote rapid recruitment of neutrophils and inflammatory monocytes; neutrophil reactive oxygen species (ROS), proteases, matrix metalloproteinases (MMPs), and neutrophil extracellular trap (NET)-associated programs amplify early inflammation and shape subsequent leukocyte waves. Recruited monocytes differentiate into macrophages that initially adopt inflammatory “M1-like” outputs (e.g., TNF, IL-1β, IL-6) and later transition toward pro-resolving/repair “M2-like” states (e.g., IL-10, TGF-β) supported by efferocytosis and tissue repair programs; in parallel, antigen presentation supports adaptive responses (Th1/Th17/Th2 and regulatory T cell polarization) that can either sustain injury or promote resolution. With chronic injury, persistent myeloid and T cell effector programs reinforce maladaptive remodeling: in arteries, these pathways contribute to plaque inflammation/necrotic core formation and to hypertensive vascular remodeling with extracellular matrix changes illustrated as elastin degradation and collagen deposition; in the myocardium, sustained immune inputs and fibroblast/myofibroblast activation are shown as scarring/fibrosis and, in some contexts, antibody-associated mechanisms. Abbreviations: Ang, angiotensin; ATLOS, artery tertiary lymphoid organs; AR, adrenergic receptor; BBB, blood–brain barrier; CVD, cardiovascular disease; DAG, diacylglycerol; DAMPs, damage-associated molecular patterns; DC, dendritic cell; DOCA, deoxycorticosterone acetate; EPAC, exchange proteins directly activated by cAMP; EC, endothelial cell; ECM, extracellular matrix; Epi, epinephrine; HF, heart failure; HFpEF, heart failure with preserved ejection fraction; HFrEF, heart failure with reduced ejection fraction; HTN, hypertension; IFNγ, interferon gamma; IL, interleukin; iNOS, inducible nitric oxide synthase; IP3, inositol triphosphate; I/R, ischemia/reperfusion; MAPK, mitogen-activated protein kinase; MI, myocardial infarction; MMPs, matrix metalloproteinases; NE, norepinephrine; NETs, neutrophil extracellular traps; PK, protein kinase; PL, phospholipase; SNS, sympathetic nervous system; TGF-β, transforming growth factor beta; Th, T helper; Treg, regulatory T cell; ROS, reactive oxygen species; TNF, tumor necrosis factor; VSMCs, vascular smooth muscle cells.

**Figure 3** summarizes the shared immune effector pathways that promote inflammatory remodeling across target organs in hypertension, including the vasculature and myocardium, linking leukocyte cytokine/ROS programs to endothelial dysfunction, ECM remodeling, and fibrosis.

### 4.2 Atherosclerosis and atherosclerotic vascular disease

Atherosclerosis is driven by chronic arterial-wall inflammation initiated by lipid retention and endothelial dysfunction, followed by recruitment of monocytes that differentiate into macrophages and foam cells, antigen-driven and innate-like T cell responses, and contributions from B cell subsets (Cybulsky and Gimbrone, 1991; Zhou et al., 2006; Hermansson et al., 2010; Kyaw et al., 2011). Adrenergic signals may arise from direct local neural inputs in the vessel wall and plaque-adjacent adventitia, from circulating catecholamines, or indirectly through neuroimmune regulation of splenic and marrow compartments before leukocytes enter the plaque (Dutta et al., 2012; Devi et al., 2021).

Direct local neuroimmune signaling is particularly relevant in advanced atherosclerosis, where adventitial remodeling and plaque-adjacent immune niches can support close interaction between nerves and immune cells (Mohanta et al., 2014; Hu et al., 2015; Ortona et al., 2026). In this context, artery tertiary lymphoid organs (ATLOs) neighboring advanced plaques provide a plausible site at which local neural and immune signals intersect (Mohanta et al., 2014; Hu et al., 2015; Ortona et al., 2026).

Sympathetic activation can therefore influence plaque biology not only through vascular tone and systemic metabolism, but also through local adrenergic signaling in immune cells that regulate chemotaxis, cytokine programs, and efferocytosis within plaques (Kojima et al., 2017; Agac et al., 2018; Hinterdobler et al., 2021a). At the same time, atherosclerosis is not regulated by sympathetic pathways alone. Indirect autonomic control through the parasympathetic–sympathetic interface, particularly the vagus–splenic axis, has been reported to modulate T cells, B cells, and macrophages in splenic compartments (Rosas-Ballina et al., 2008; Rosas-Ballina et al., 2011; Kooijman et al., 2015). This suggests that atherosclerotic inflammation may be shaped both by direct plaque-adjacent neuroimmune signaling and by remote autonomic control of leukocyte priming and mobilization. This distinction should be made explicitly whenever adrenergic mechanisms in atherosclerosis are discussed. Because lesion development is prolonged, chronic sympathetic activation is more likely to influence long-term leukocyte recruitment, receptor responsiveness, and inflammatory tone, whereas acute sympathetic surges (e.g., stress, infection, MI) may transiently reprogram leukocyte recruitment and effector function within plaques and at sites of plaque disruption (Hinterdobler et al., 2021a; Hinterdobler et al., 2021b). Thus, acute and chronic adrenergic effects should not be treated as interchangeable. Available studies suggest that β_2_AR–cAMP signaling can restrain certain inflammatory functions in myeloid cells, whereas α-adrenergic pathways may promote adhesion and inflammatory activation in specific contexts; however, conclusions are often limited by systemic drug delivery and the difficulty of disentangling immune-intrinsic effects from changes in hemodynamics and lipid handling (Marrett et al., 1986; Grisanti et al., 2011b; Hinterdobler et al., 2021b). Accordingly, the current evidence supports adrenergic regulation of atherosclerotic inflammation, but it does not yet fully resolve which effects are plaque-local, which reflect circulating catecholamines, and which arise indirectly through splenic or marrow pathways.

The role of macrophage polarization in atherosclerosis also requires more precise wording than a simple M1/M2 binary (Mantovani et al., 2009; Moore et al., 2013; Peled and Fisher, 2014). Reparative or M2-like macrophage features are generally associated with anti-inflammatory cytokine production, efferocytosis, and resolution programs that would be expected to support plaque stability rather than progression (Peled and Fisher, 2014; Kojima et al., 2017). However, macrophages with reparative features can still accumulate lipid within plaques, and phagocytic activity should not be equated uniformly with protection (Mantovani et al., 2009; Moore et al., 2013). Clearance of apoptotic cells (efferocytosis) is generally protective and limits necrotic-core expansion, whereas uptake of modified lipoproteins promotes foam-cell formation (PMID: 28137963, 23995626). Thus, macrophage phagocytosis has context-dependent consequences, and M2-like properties should not be interpreted as simply anti-atherosclerotic in all settings (Moore et al., 2013) (Peled and Fisher, 2014).

Taken together, atherosclerosis is one of the clearest examples in which the manuscript must distinguish among direct local neural signaling, circulating catecholamine exposure, and indirect autonomic regulation through lymphoid organs (Mohanta et al., 2014; Hu et al., 2015; Devi et al., 2021). It is also the section in which sympathetic–parasympathetic interactions should be acknowledged explicitly (Vida et al., 2011; Heusch and Kleinbongard, 2025). Immune-cell–specific receptor deletion in atherosclerosis-prone models and single-cell, plaque-resolved receptor mapping remain key needs for the field (Depuydt et al., 2020).

### 4.3 Acute MI and ischemia–reperfusion injury

Acute MI and ischemia–reperfusion (I/R) injury trigger a massive catecholamine surge on top of sterile inflammation from cardiomyocyte necrosis (Bertel et al., 1982; Nahrendorf et al., 2007). In contrast to chronic vascular disease, adrenergic signals in MI arise both from circulating catecholamines and from local cardiac sympathetic nerve terminals within or adjacent to the injured myocardium. NE and Epi are released systemically and from cardiac sympathetic nerve terminals, engaging ARs on neutrophils, monocytes, macrophages, dendritic cells, and lymphocytes infiltrating the infarcted myocardium (Lameris et al., 2000; Grisanti et al., 2016a).

Neutrophils are early responders whose functions are tuned by adrenergic input (Garcia-Prieto et al., 2017; Marino et al., 2018). β_2_AR–Gs–cAMP signaling generally restrains chemotaxis, ROS/degranulation, and NETosis, whereas inducible α_1_AR signaling can favor integrin-dependent adhesion and pro-inflammatory activation, exacerbating oxidative injury (Silvestri et al., 1999; Grisanti et al., 2011a). Across acute sterile inflammation models—including MI/I/R—β_2_AR-linked programs can shape both the magnitude and resolution of neutrophil responses (including recruitment kinetics, persistence, and clearance (Kim et al., 2014; Garcia-Prieto et al., 2017). Where mechanistic evidence is drawn from non-cardiac tissues or systemic stress paradigms, this extrapolation should be stated explicitly and validated in MI-specific, neutrophil-intrinsic studies (Kim et al., 2014).

Monocyte and macrophage dynamics are likewise governed by ARs (Grisanti et al., 2011b; Grisanti et al., 2016a). After MI, CCR2+ classical monocytes are mobilized from bone marrow and spleen and recruited to the infarct, where they differentiate into inflammatory and subsequently reparative macrophages (Nahrendorf et al., 2007). This is therefore another setting in which both local cardiac adrenergic signals and indirect marrow/splenic neuroimmune effects likely contribute. Leukocyte-expressed β_2_AR is essential for proper early inflammatory repair. Mice lacking β_2_AR on leukocytes show altered leukocyte trafficking, impaired scar formation, and increased risk of rupture in MI models (Grisanti et al., 2016a; Grisanti et al., 2016b). β_2_AR–cAMP signaling in macrophages promotes efferocytosis, anti-inflammatory cytokine production, and matrix remodeling, but under some conditions, β_2_AR loss can blunt maladaptive remodeling in chronic settings, underscoring strong context dependence (Negreiros-Lima et al., 2020; Nayak et al., 2026). α_1_AR and α_2_AR on myeloid cells may also contribute by modulating vasoconstriction, platelet–leukocyte interactions, and microvascular adhesion, influencing no-reflow and microvascular obstruction, though immune cell–specific *in vivo* data remain limited (Michaels et al., 2000; Grisanti et al., 2011a).

Overall, adrenergic signaling in innate immune cells in MI affects: (1) infarct size and cardiomyocyte survival by tuning cytotoxic neutrophil activity (Garcia-Prieto et al., 2017); (2) clearance of dead cells and debris via β_2_AR-modulated efferocytosis (Nayak et al., 2026); and (3) scar formation via control of inflammatory-to-reparative macrophage transitions and downstream fibroblast activation (Grisanti et al., 2016a; Garcia-Prieto et al., 2017; Nayak et al., 2026). MI is thus a prototypical setting where catecholamine surges and immune-cell AR signaling are tightly intertwined. At this acute stage following MI, adrenergic regulation is often adaptive. Short, pulsatile catecholamine bursts can help coordinate leukocyte trafficking, limit collateral injury, and support timely transition toward resolution and repair (Grisanti et al., 2016a; Grisanti et al., 2016b). Where mechanistic details are inferred from studies in non-cardiac tissues or systemic inflammation models, extrapolation to the infarct niche should be stated explicitly; the strongest support comes from MI/I/R studies that combine immune-cell-specific receptor perturbation with*in situ* functional readouts (Grisanti et al., 2016a; Nayak et al., 2026).

### 4.4 Chronic heart failure (ischemic and non-ischemic, including pressure overload)

Chronic HF is characterized by persistent SNS activation and elevated NE, which correlates with worse outcomes (Francis et al., 1993; Joseph and Gilbert, 1998). Catecholamines continuously stimulate ARs not only on cardiomyocytes and vascular cells but also on resident and infiltrating immune cells, macrophages, dendritic cells, mast cells, T cells, and B cells, in the failing heart (Levick and Widiapradja, 2018; Strassheim et al., 2019). In chronic HF, adrenergic input is likely sustained through both local cardiac sympathetic activity and circulating catecholamines, with additional indirect effects on immune-cell production and mobilization potentially arising through marrow and splenic compartments (Joseph and Gilbert, 1998; Hulsmans et al., 2018).

A key consequence of prolonged catecholamine exposure in chronic HF is the potential decoupling of high sympathetic tone from its immunoregulatory functions (Sharma and Farrar, 2020; Tanner et al., 2021). Desensitization or downregulation of β_2_AR can blunt cAMP–PKA-dependent suppression of pro-inflammatory mediators and weaken pro-resolving programs (including IL-10-linked pathways and efferocytosis), while persistent α-adrenergic inputs and/or pathway reweighting (e.g., Gαi/MAPK-leaning outputs) may favor leukocyte survival, recruitment, and, in some contexts, pro-fibrotic signaling (Wahle et al., 2001; Tan et al., 2007; Tanner et al., 2021). Together, these changes provide a mechanistic framework for how time-limited adaptive inflammation can transition into self-sustaining low-grade inflammation, fibrosis, and microvascular dysfunction in advanced HF (Hulsmans et al., 2018; Tanner et al., 2021).

In this setting, adrenergic receptor regulation becomes a mechanistic variable rather than a background detail: sustained catecholamine drive can actively remodel receptor availability and coupling via GRK/β-arrestin–dependent phosphorylation, desensitization, and internalization (classically described for cardiac β_1_AR) (Ungerer et al., 1993; Ungerer et al., 1994). If similar regulatory programs occur in leukocytes, chronic HF would be expected to dampen β_2_AR–cAMP/PKA anti-inflammatory signaling and shift therapeutic responsiveness; however, immune-cell–intrinsic *in vivo* evidence for this desensitization axis in HF remains limited and warrants targeted investigation (Agac et al., 2018; Tanner et al., 2021).

Macrophages expand and diversify in pressure overload and HF (including HFpEF), with both CCR2+ recruited and resident populations contributing to chronic inflammation, fibroblast activation, and microvascular dysfunction (Hulsmans et al., 2018; Patel et al., 2018). These macrophages express β_2_AR, α_1_AR, and α_2_AR (Ignatowski et al., 2000; Tan et al., 2007; Grisanti et al., 2011b); chronic catecholamine exposure can drive IL-6, TGFβ, and profibrotic programs directly in macrophages or indirectly via fibroblast and endothelial crosstalk (Tan et al., 2007; Tanner et al., 2021). Importantly, immune cell β_2_AR has been shown to be required for full development of HF phenotypes in a chronic isoproterenol model. Mice with immune-specific β_2_AR deletion have markedly reduced proinflammatory macrophage infiltration, cardiomyocyte death, hypertrophy, fibrosis, and improved function (Tanner et al., 2021). These data identify immune β_2_AR as a critical amplifier of catecholamine-driven remodeling (Tanner et al., 2021).​.

B cells in HF produce cytokines (IL-6, IL-10) and autoantibodies against β_1_AR, muscarinic receptors, myosin, and other cardiac antigens; such autoantibodies can directly modulate receptor function, trigger arrhythmias, and drive dilated remodeling (Jahns et al., 2004; Jane-wit et al., 2007). Clinical data link β_1_AR autoantibodies with worse LV function and outcomes, and selective removal can improve function (Stork et al., 2006; Dandel et al., 2012). Chronic adrenergic stress may influence shapes germinal center dynamics, Breg vs pathogenic B cell balance, and autoantibody repertoires, although mechanistic details are still emerging (Sanders, 2012; Honke et al., 2022).

These immune AR programs interact closely with cardiac fibroblasts and endothelium. Macrophage- and T cell–derived IL-6, IL-1β, TNF, TGFβ, and chemokines drive fibroblast activation, collagen deposition, and microvascular rarefaction (Ma et al., 2012; Nevers et al., 2017), while endothelial cells respond to catecholamines and cytokines by upregulating adhesion molecules and chemokines, sustaining leukocyte recruitment (Devaux et al., 1997; Kapper et al., 2002). In HF, ARs on immune cells thus act as neuro-immune amplifiers that couple persistent SNS activation to chronic inflammation, fibrosis, and progressive pump failure (Hulsmans et al., 2018; Tanner et al., 2021).

As in other CVD settings, mechanistic clarity is limited by species differences and by reliance on systemic adrenergic manipulation. Studies that combine immune-cell–specific AR perturbation with cardiovascular-relevant endpoints (remodeling progression, fibrosis, function, and immune composition) will be important for defining therapeutic windows and minimizing off-target risk (Grisanti et al., 2016a; Tanner et al., 2021).

## 5 Translational considerations and therapeutic opportunities

Current evidence supports the idea that AR signaling in immune cells may represent a therapeutically relevant axis in CVD, but most data remain indirect and do not yet justify strong claims about near-term immune-targeted AR therapy (Sharma and Farrar, 2020; Nayak et al., 2024). In practice, the clearest translational signal still comes from existing systemic AR-active drugs, especially β-blockers, which improve cardiovascular outcomes while also being associated with changes in inflammatory and immune phenotypes, although these effects cannot be cleanly separated from hemodynamic and neurohumoral actions (CIBIS-II Investigators and Committees, 1999; von Haehling et al., 2009). Looking forward, one attractive direction is the development of biased ligands that preferentially engage signaling branches linked to beneficial outcomes, for example, preserving pro-resolving or anti-inflammatory programs while limiting maladaptive remodeling signals, an idea supported conceptually by agents such as carvedilol and nebivolol, though immune-cell–intrinsic validation in CVD remains limited (Noma et al., 2007; Erickson et al., 2013). A second goal is greater receptor subtype and cell-type specificity, since the same AR subtype can be adaptive in one compartment yet maladaptive in another; accordingly, future strategies may need to target defined immune lineages or tissue niches rather than rely on global agonism or blockade, but such approaches remain largely preclinical and face major delivery and safety challenges (Chhatar and Lal, 2021; Udriste et al., 2024). For now, the most realistic clinical framework is to view adrenergic immunomodulation as a potential adjunct to established therapies, including β-blockers, RAAS inhibitors, SGLT2 inhibitors, MRAs, and statins, rather than a replacement for standard care, with the key next step being to determine whether immune-focused AR modulation can add benefit beyond existing guideline-directed treatment in disease- and stage-specific settings (Heidenreich et al., 2022; Markousis-Mavrogenis et al., 2024).

## 6 Knowledge gaps and future directions

Despite growing interest in adrenergic regulation of immune cells in CVD, several major gaps still limit mechanistic interpretation and translational progress. First, AR expression across immune subsets remains incompletely mapped in human CVD. Most detailed information on α_1_-, α_2_-, β_1_-, β_2_-, and β_3_AR expression comes from murine models or *in vitro* studies of human leukocytes under non-physiological conditions, whereas systematic profiling across major immune lineages directly in human vessels, perivascular tissues, lymphoid compartments, and diseased hearts is still sparse (Litvinukova et al., 2020; Chhatar and Lal, 2021). Addressing this gap will require integrated single-cell and spatial approaches, coupled with high-dimensional proteomics and rigorous validation of probes and antibodies, because GPCR transcripts are often low abundance and available protein-detection tools can be unreliable (Jia et al., 2018; Insel et al., 2019). Just as importantly, future studies need to resolve context: AR expression and function are likely to differ across acute versus chronic disease, ischemic versus non-ischemic settings, HFpEF versus HFrEF, and across comorbid states such as obesity, diabetes, chronic kidney disease, and aging (Sweet et al., 2018; Smart and Madhur, 2023). These studies should also more clearly distinguish direct local norepinephrine exposure in cardiovascular tissues from circulating catecholamines and indirect neuroimmune regulation through spleen and bone marrow, rather than treating these sources as interchangeable (Carnagarin et al., 2019; Ahmari et al., 2020).

A second major priority is mechanistic dissection of subtype- and cell-specific AR functions *in vivo*. Many current conclusions still rely on global knockouts, incompletely selective pharmacology, or reductionist *in vitro* systems that lack tissue context (Wang et al., 2021; Nayak et al., 2024). More precise genetic and pharmacologic approaches will be needed to define how α_1_-, α_2_-, β_1_-, β_2_-, and β_3_AR signaling in myeloid cells, T cells, B cells, and other immune populations shapes inflammation, repair, and remodeling in hypertension, atherosclerosis, MI, and HF (Grisanti et al., 2016a; Honke et al., 2022). These efforts should move beyond receptor presence alone and test how specific signaling branches contribute to disease, including G-protein– versus β-arrestin–linked pathways, ideally using immune cell–restricted perturbations, adoptive transfer, intravital imaging, fate mapping, and multi-omic profiling (Liggett, 2011; Urban and Roth, 2015). Likewise, evaluation of biased ligands and β-arrestin–biased β-blockers should include explicit immune readouts, rather than focusing only on hemodynamics or ventricular function (Kim et al., 2020; Chen et al., 2025).

Finally, translation will require better biomarkers and trial designs that capture immune effects directly. At present, there are no validated clinical biomarkers of AR signaling status in immune cells in cardiovascular patients. Potential approaches include flow or mass cytometry panels for AR expression, GRKs, and β-arrestin-related markers; ex vivo assays of catecholamine responsiveness in patient leukocytes; and imaging strategies that relate sympathetic activity or receptor status to vascular or myocardial inflammation (Saygin et al., 2018; Zelt et al., 2020). Future trials of β-blockers, α-blockers, biased ligands, neuromodulatory strategies, or AR-targeted delivery systems should incorporate immune phenotyping as a predefined component, including circulating and tissue-resident immune subsets, cytokine programs, autoantibody profiles, and imaging markers of inflammation (Panahi et al., 2018; Markousis-Mavrogenis et al., 2024). Overall, the field now needs fewer broad assumptions and more integrated human mapping, immune cell–specific mechanistic testing, and translational frameworks that determine whether immune-cell adrenergic signaling can be monitored and therapeutically leveraged in a disease- and stage-specific manner.

## 7 Conclusion

ARs on immune cells form an important interface between sympathetic stress and cardiovascular inflammation. Through α_1_, α_2_, and β-adrenergic receptors, leukocytes continuously “sense” catecholamine tone and translate it into changes in cytokine production, chemotaxis, effector function, and tissue remodeling, thereby linking neurohumoral activation to vascular injury and remodeling and to cardiac injury, repair, and chronic remodeling (Chhatar and Lal, 2021; Nayak et al., 2024).

All AR subtypes contribute to this interface in a cell-type- and context-dependent manner (Sharma and Farrar, 2020; Chhatar and Lal, 2021). α-adrenergic signaling often favors vasoconstriction, leukocyte adhesion, and pro-inflammatory activation, whereas β-adrenergic signaling more commonly restrains acute inflammation and promotes resolution (Grisanti et al., 2011b; Agac et al., 2018), yet can also facilitate detrimental macrophage infiltration and fibrosis in chronic HF, depending on disease stage and receptor remodeling (Tanner et al., 2021). Plasticity in receptor density, G-protein coupling, and subcellular localization, together with biased agonism and desensitization, helps explain why the same receptor can be protective in acute vascular injury and MI—by enabling appropriate leukocyte recruitment and repair—and harmful in chronic catecholamine excess, by sustaining pro-inflammatory macrophage influx and adverse remodeling (Grisanti et al., 2016b; Wu et al., 2018; Tanner et al., 2021). These insights support further investigation into therapies that move beyond simple “on–off” AR blockade toward precision targeting of adrenergic signaling in immune cells. By combining subtype-specific and cell-specific strategies with biased ligands that favor pro-resolving over pro-fibrotic pathways, it may become possible to harness adrenergic signaling as a tunable checkpoint: dampening chronic cardiovascular inflammation and fibrosis while preserving essential host defense and repair functions (Chhatar and Lal, 2021; Nayak et al., 2024).
